# Rapid changes in serum cytokines and chemokines in response to inactivated influenza vaccination

**DOI:** 10.1111/irv.12509

**Published:** 2018-01-04

**Authors:** Kawsar R. Talaat, Neal A. Halsey, Amber B. Cox, Christian L. Coles, Anna P. Durbin, Amritha Ramakrishnan, Jay H. Bream

**Affiliations:** ^1^ Center for Immunization Research (CIR) Johns Hopkins Bloomberg School of Public Health Baltimore MD USA; ^2^ Institute for Vaccine Safety Department of International Health Johns Hopkins Bloomberg School of Public Health Baltimore MD USA; ^3^ Infectious Disease Clinical Research Program Uniformed Services University of the Health Bethesda MD USA; ^4^ Department of Molecular Microbiology and Immunology Johns Hopkins Bloomberg School of Public Health Baltimore MD USA

**Keywords:** chemokines, cytokines, inactivated, inactivated vaccine, influenza vaccine, symptoms

## Abstract

**Background:**

The timing of host cytokine responses to influenza vaccination is poorly understood.

**Objectives:**

We examined serum cytokine kinetics following inactivated trivalent influenza vaccine (TIV) to better understand potential relationships between markers of inflammation and TIV‐related side effects.

**Patients/Methods:**

Twenty healthy adult subjects received TIV. Cytokines/chemokines were assessed in intervals from 3 hours to 14 days. Antibody titers were measured at baseline and Day 14.

**Results:**

Serum cytokine responses to TIV were evident as early as 3 hours post‐immunization. Compared to baseline, IFN‐γ and IP‐10 were significantly elevated 7 hours after TIV administration. Both remained elevated and peaked between 16 and 24 hours before returning to baseline by 44 hours post‐vaccination. Although IL‐8 levels were variable between subjects during the first 24 hours after TIV, by 44 hours, IL‐8 was significantly lower compared to baseline. Interestingly, IL‐8 levels remained significantly lower for up to 2 weeks after receiving TIV. Fifteen of 20 subjects reported mild adverse events. The one subject who reported moderate myalgias and injection site pain after vaccination displayed a distinctive, early cytokine response profile which included IL‐6, IL‐2, IL‐8, IP‐10, MCP‐1, TNF‐α, TARC, and MCP‐4.

**Conclusions:**

Serum cytokines changed rapidly following TIV and generally peaked at 24 hours. Trivalent influenza vaccine‐induced reductions in IL‐8 occurred later (44 hours) and were sustained for 2 weeks. An outlier response coincided with the only moderate side effects to the vaccine. These data suggest that early cytokine/chemokine responses may provide additional insight into the pathogenesis of adverse events and immune reactivity to vaccination.

## 1 INTRODUCTION

Cytokines and chemokines play an integral, yet somewhat paradoxical role in host defense against influenza. For example, type I interferons have strong antiviral activities and can directly inhibit influenza virus replication.^1^, ^2^ Meanwhile, excessive cytokine/chemokine responses have been associated with more severe disease during the 2009 H1N1 pandemic,^3^ lung damage in macaques infected with the 1918 influenza virus,^4^ and fatal H5N1 infection in humans.^5^

The role of cytokines in influenza vaccine responses is less clear. In the case of the smallpox vaccine, cytokines are linked not only with vaccine efficacy but also with adverse events.^6^ A frequently cited rationale for avoiding annual influenza vaccines is concern about experiencing side effects.^7^, ^8^, ^9^, ^10^, ^11^ Public concerns about vaccine side effects can undermine immunization programs, including national or statewide seasonal influenza vaccine campaigns.^12^, ^13^ Although local and systemic adverse events are generally transient and short‐lived after influenza vaccines,^14^ predictable post‐vaccine reactogenicity events like myalgia and malaise in the first 2 days after influenza vaccination have led many to the misperception that the vaccine “gave them the ‘flu’”.^15^, ^16^ Although this phenomenon has been well described,^16^ there are little data to explain the biologic basis of these events and the relationship (if any) with cytokine responses.

Recently, systems biology approaches have been used to prospectively explore the molecular determinants of influenza vaccine responses including efficacy and/or adverse events.^17^, ^18^ Although different vaccines were used, both of these studies identified early immune gene expression signatures (1‐3 days after immunization) which predicted immunogenicity^17^ and the onset of clinical adverse events.^18^

In line with these data, another report described an association between serum cytokines and subjective side effects in women 1‐2 days after receiving TIV.^19^

As the role of cytokines in vaccine responses continues to be defined, we sought to more discretely characterize early serum cytokine kinetics in response to TIV in this proof‐of‐principle study. The study included 2 groups of vaccinees (n = 10/group) that collectively were assessed at baseline and 3 hours, 7 hours, 16 hours, 24 hours, 44 hours, and 14 days after vaccination for serum cytokines/chemokines, hemagglutination inhibition (HI) titers, and subjective side effects.

## 2 METHODS

### 2.1 Study design

This was an open‐label study. The study was approved by the Johns Hopkins Bloomberg School of Public Health Institutional Review Board (IRB) and was conducted in accordance with the principles of the Declaration of Helsinki and the Standards of Good Clinical Practice (as defined by the International Conference on Harmonisation). All participants provided written informed consent.

### 2.2 Vaccine

The 2011‐2012 licensed trivalent inactivated influenza vaccine (Fluzone^®^ Sanofi‐Pasteur, Swiftwater, PA, USA, LotUH493AA) was administered in the standard 0.5 mL dose intramuscularly in the deltoid muscle using a 7/8‐inch needle. The vaccine contained 15 μg of HA of each of following strains: an H1N1 A/California/7/2009‐like virus, an H3N2 A/Perth/16/2009‐like virus, and a B/Brisbane/6/60/2008‐like virus.

### 2.3 Participants and study procedures

Healthy, non‐pregnant adults 18‐50 years of age were recruited at the Center for Immunization Research, Johns Hopkins Bloomberg School of Public Health. Participants were excluded if they had a history of allergy to eggs or other components of the influenza vaccine, or if they had already received the 2011‐2012 seasonal influenza vaccine. Additional exclusionary criteria included any immunocompromising condition, including HIV infection, active hepatitis B or C infection, diabetes, chronic inflammatory diseases, autoimmune diseases, concurrent illness or infection, use of chronic corticosteroids, splenectomy, history of anaphylaxis, receipt of investigational product, or live vaccine within 30 days. Subjects were questioned on previous history of influenza infection, prior influenza vaccinations, and any reactions they may have had using a questionnaire. Participants were randomized using a random number generator into two groups to maximize the number of time points, and minimize inconvenience to the outpatient volunteers by avoiding nighttime blood draws. Group 1 (subjects 1‐10) received the TIV in the morning; they had blood drawn before vaccination and at 3, 7, 24, and 48 hours later. Group 2 (subjects 11‐20) received the TIV in the afternoon; they had blood drawn before vaccination and at approximately 16 and 40 hours after vaccination. Both groups had a blood draw on Day 14. No differences were noted at 40 and 48 hours, so the data were combined for analyses and assigned the time of 44 hours.

At each post‐vaccination assessment, subjects were questioned about injection site reactions (erythema, induration, swelling, pain, pruritus, ecchymosis) and systemic reactions (headache, myalgia, malaise, shivering, fever). These and other symptoms were assessed for severity, seriousness, and relationship to the influenza vaccine. Post‐vaccination assessments were modified from the current “Guidance for Industry: Toxicity Grading Scale for Healthy Adult and Adolescent Volunteers Enrolled in Preventive Vaccine Clinical Trials” (September 2007, DHHS, FDA, CBER). Adverse events were defined as mild, moderate, or severe. Mild symptoms are those that the volunteer is aware, but does not interfere with activity. Moderate symptoms may inhibit some activity and require use of medication to alleviate the symptoms. Significant interruption of daily activities, such as not being able to go to work or being sick enough to visit a doctor, is considered severe adverse event. Injection site reactions were measured in centimeters and graded according to the size of the reaction observed.

### 2.4 Assays

Hemagglutination inhibition titers were determined at baseline and Day 14 as described previously.^20^ The sera were tested for antibodies against the 3 viral strains included in the 2011‐2012 influenza vaccine: H1N1 A/California/7/2009‐like virus, H3N2 A/Perth/16/2009‐like virus, and B/Brisbane/6/60/2008‐like virus (Victoria lineage) and against B/Wisconsin/1/2010‐like virus (Yamagata lineage) not included in the vaccine.

#### 2.4.1 Multiplex cytokine assay

Serum cytokines and chemokines were measured using the Meso Scale Discovery (MSD) platform (MSD, Gaithersburg, MD, USA). The Human Proinflammatory 9‐Plex Ultra‐sensitive Kit included: GM‐CSF, IFN‐γ, IL‐10, IL‐12p70, IL‐1β, IL‐2, IL‐6, IL‐8 (CXCL8), and TNF‐α. The Human Chemokine 7‐Plex Ultra‐Sensitive Kit included: eotaxin (CCL11), IL‐8 (CXCL8), IP‐10 (CXCL10), MCP‐1 (CCL2), MCP‐4 (CCL13), MIP‐1β (CCL4), and TARC (CCL17). IL‐8 was in both panels. In addition, we measured C‐reactive protein (CRP), serum amyloid A (SAA), and the soluble cell adhesion molecules sVCAM‐1 and sICAM‐1 using the MSD Vascular Injury Panel II. Meso Scale Discovery plates were analyzed on the SECTOR Imager 2400 as previously described.^21^ All samples were run in duplicate.

### 2.5 Statistics

Statistical analyses were performed using Prism software v4.0c (GraphPad, San Diego, CA, USA) and STATA (VERSION) (StataCorp, College Station, TX, USA). Differences in median cytokine levels were assessed using the Wilcoxon signed rank test. Corrections were not made for multiple comparisons because this is a pilot study and we wanted to explore potential signals. The baseline statistics for each cohort was summarized using a t‐test for continuous data and chi‐square for contingency data. For the dynamics of the cytokine response, a nonparametric test was performed to look at paired data on selected cytokines. Non‐responder values were assigned the lower limit of quantitation value.

Cohorts were compared in terms of baseline characteristics (socioeconomic status, demographics, antibody titers at baseline and at 14 days, and the number of non‐responders).

## 3 RESULTS

### 3.1 Demographics

**Figure 1:**
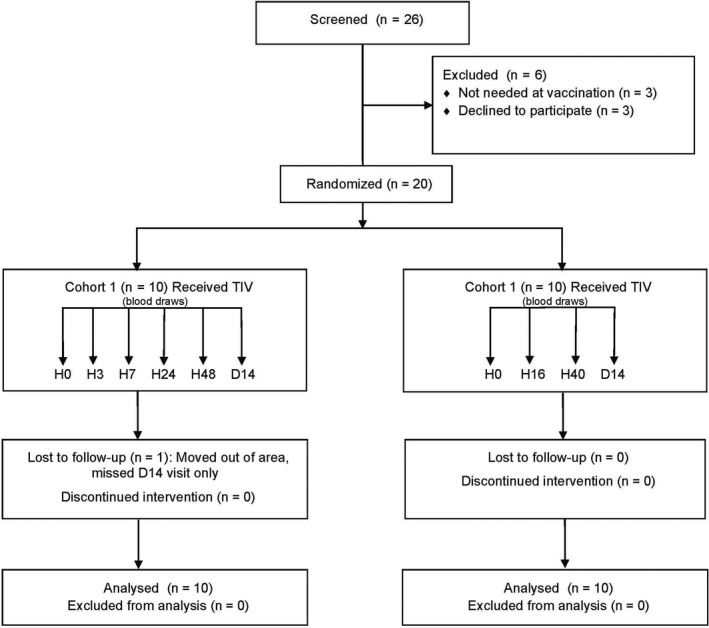
Consort Diagram. Screening and enrollment of subjects and allocation by cohort. Abbreviations: TIV, trivalent inactivated influenza vaccine; H, hours; D, days; n, number.

This study was conducted in January and February 2012. Twenty‐six subjects were screened and consented (Figure 1). Twenty subjects received TIV. Baseline characteristics of the study participants are shown in Table 1. The average age for all participants was 37 years old, with a range from 24 to 48 years. Nine of 20 subjects were female and all except two were Black, reflecting our population in East Baltimore. The only difference between cohorts 1 and 2 was in gender—in Cohort 1, 70% of subjects were female, compared with 20% in Cohort 2 (*P* = .03 Wilcoxon signed rank test). One subject in Group 1 did not return for the Day 14 visit. The remaining subjects completed all scheduled visits, and all subjects were included in the analysis. Of the eight subjects who had previously received influenza vaccination, only 2 subjects (subjects 14 and 16) had been vaccinated the prior season.

**Table 1:** Characteristics of study participants

Characteristic	Cohort 1 (n = 10)		Cohort 2 (n = 10)		*P*‐value
	n		n		
Female (%)	7	70	2	20	.03
Age category (%)					
21‐29 y	4	40	4	40	.80
30‐39 y	1	10	2	20	
40‐49 y	5	50	4	40	
Mean age (mean [SD]) y		38.1 (9.4)		35.8 (10.1)	.60
Ethnicity (%)					
Black	10	100	8	80	.14
White	0	0	2	20	
Hx of Flu (%)	0	0	1	10	.31
Flu vaccination in past 10 y (%)	5	50	3	30	.36

### 3.2 Antibody responses to trivalent influenza vaccine

Approximately 90% of the subjects had a ≥4‐fold increase in HI titers at Day 14 to each of the three antigens included in the vaccine (Table 2). Responses in the 2 cohorts were comparable to all 4 antigens tested. Most subjects had a ≥4‐fold increase in HI titer to the B/Wisconsin strain that was not included in the vaccine, suggesting cross‐reactivity between the 2 influenza B strains. The 2 volunteers who had received the previous year's vaccination, subjects 14 and 16, had smaller fold increases in HI titer than did those who did not. Subject 14, who had pre‐existing titers to most of the strains prior to vaccination, had no response to the H3N2 component and only a twofold response to both B strains. Subject 16 had a twofold response to the H3N2 component and fourfold to the other strains. Subject 8, who had the most robust cytokine responses, had a 32‐ to 64‐fold increase in HI titer to the 3 strains in the influenza vaccine at 2 weeks. The strong antibody response to the vaccine components and the unrelated influenza B strain suggested a memory response to the vaccine.

**Table 2:** Hemagglutination inhibition (HI) responses to trivalent influenza vaccine

Virus	GM HI Titer		Fold increase (SD)
	Baseline (SD)	d14 (SD)	
H1N1 A/California/7/2009‐like^a^	22 (157)	1185 (3007)	51 (149)
H3N2 A/Perth/16/2009‐like^a^	21 (225)	247 (597)	12 (39.7)
B/Brisbane/6/60/2008‐like^a^	5.3 (14)	119 (112)	25 (46)
B/Wisconsin/1/2010‐like	3.6 (7)	69 (77)	21 (42)

a Viruses included in the 2011‐2012 trivalent seasonal vaccine.

### 3.3 Cytokine/chemokine responses to trivalent influenza vaccine

**Figure 2:**
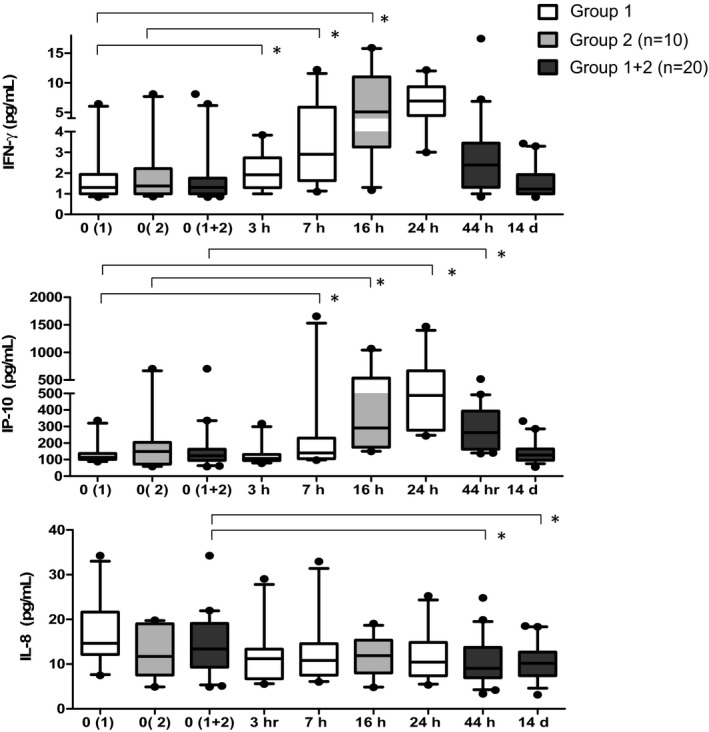
Serum cytokine levels in TIV recipients. Time course of serum IFN‐γ, IP‐10, and IL‐8 levels in TIV vaccine recipients at 0 h (prior to vaccination), 3 h, 7 h, 16 h, 24 h, 44 h, and 14 d post‐vaccination. Participants in group 1 ( 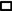 ) had serum drawn at 0 h, 3 h, 7 h, 16 h, 24 h, 44 h, and 14 d post‐vaccination. Participants in group 2 ( 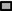 ) had serum drawn at 0 h, 16 h, 44 h, and 14 d post‐vaccination. Combined cytokine levels for groups 1 and 2 at 0 h, 44 h, and 14 d are also shown ( 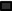 ). The top and bottom of the box represent the 75th and 25th percentiles, respectively. The line near the middle of the box represents the median (50th percentile) and ends of the whiskers are drawn to the 10th and 90th percentiles. *P < .05, comparing pre‐ and post‐vaccination levels within each group. The Wilcoxon signed rank test was used to determine *P*‐values. Abbreviations: TIV, trivalent inactivated influenza vaccine; h, hours; d, days; n, number.

Compared to baseline values, significant differences in median cytokine/chemokine levels were noted at one or more time points for all of the proinflammatory and chemokine analytes tested with the exception of eotaxin (Table 3). However, it was difficult to categorize patterns of change over time for many of the analytes due to considerable interindividual variability. IFN‐γ, IP‐10, and IL‐8 had distinct temporal profiles with considerable overlap between the groups (Figure 2, Table 3). Significant increases in IFN‐γ occurred by 7 hours and peaked between 16 and 24 hours before returning to baseline by 44 hours post‐vaccination. Similarly, IP‐10 also increased significantly by 7 hours, with a peak at 24 hours, declining at 44 hours, with a return to baseline by Day 14. No significant differences in IL‐8 were observed at the earliest time points, but at 44 hours post‐vaccination, IL‐8 levels were decreased compared to baseline (Figure 2, Table 3). As we reported previously, IL‐8 remained low at Day 14.^22^

**Table 3:** Changes in serum cytokines following vaccination with trivalent influenza vaccine

Cytokine	Median (SD) Cytokine level pg/mL								
	Group 1				Group 2		Groups 1 and 2^a^		
	0 h	3 h	7 h	24 h	0 h	16 h	0 h	44 h	14 d
IFN‐γ	1.3 (1.7)	1.9 (1.0)	**2.9 (3.4)** b	**6.9 (3.0)** b	1.4 (2.2)	**5.1 (5.0)** b	1.3 (1.9)	2.4 (3.7)	1.2 (0.8)
IL‐2	0.7 (0.3)	0.7 (1.2)	**1.1 (3.6)** b	**0.9 (0.6)** b	0.6 (0.3)	0.9 (0.3)	0.7 (0.3)	0.7 (0.3)	0.7 (0.3)
IL‐6	2.3 (1.9)	2.0 (0.8)	3.3 (10.8)	2.9 (1.3)	2.1 (18.2)	2.5 (15.8)	2.3 (12.9)	**1.6 (7.2)** b	**1.6 (7.5)** b
IL‐8	14.7 (7.7)	11.2 (6.9)	10.8 (7.9)	10.4 (6.1)	11.7 (5.5)	11.9 (4.6)	13.4 (6.9)	**9.0 (5.4)** b	**10.2 (4.1)** b
GM‐CSF	0.9 (1.1)	0.9 (1.0)	0.8 (1.1)	1.0 (0.8)	1.0 (0.4)	1.0 (0.4)	0.9 (0.8)	**0.8 (0.8)** b	1.0 (0.8)
IP‐10	115 (74)	106 (69)	**140 (482)** b	**488 (365)** b	149 (194)	**292 (309)** b	123 (146)	**263 (127)** b	127 (71)
TARC	995 (429)	1028 (462)	**1079 (567)** b	1054 (3072)	871 (993)	998 (1013)	970 (757)	1000 (1667)	901 (555)
MCP‐4	760 (306)	796 (302)	**840 (359)** b	858 (803)	881 (295)	814 (267)	863 (297)	787 (288)	836 (233)
MCP‐1	378 (160)	**315 (142)** b	389 (897)	**372 (282)** b	334 (315)	372 (190)	349 (247)	324 (147)	**320 (171)** b
Eotaxin	853 (1159)	909 (987)	1000 (1204)	860 (1190)	999 (1133)	959 (792)	901 (1116)	783 (757)	1017 (1011)
IL‐12p70	2.6 (94.9)	3.1 (118)	3.1 (116)	**3.1 (134)** b	1.3 (2.0)	**1.9 (1.8)** b	2.0 (67.4)	2.3 (83.7)	2.2 (101)
TNF‐α	6.9 (3.7)	8.4 (3.3)	8.4 (4.2)	**9.5 (2.7)** b	9.1 (3.5)	8.7 (2.3)	7.8 (3.5)	8.1 (2.2)	7.8 (2.1)
IL‐10	5.4 (158)	**6.0 (214)** b	**6.7 (209)** b	**7.8 (241)** b	3.1 (2.6)	4.7 (1.4)	4.5 (112)	4.9 (151)	4.9 (149)
MIP1β	123 (85)	133 (89)	**145 (92)** b	**177 (77)** b	147 (102)	173 (94)	141 (92)	145 (82)	136 (71)

a Data are combined from groups 1 and 2. Values in bold are have *P*<.05 compared with 0‐h time point.

b *P* < .05 compared with 0‐h time point in each group; Wilcoxon signed rank test. SD, standard deviation; h, hours; d, days.

**Figure 3:**
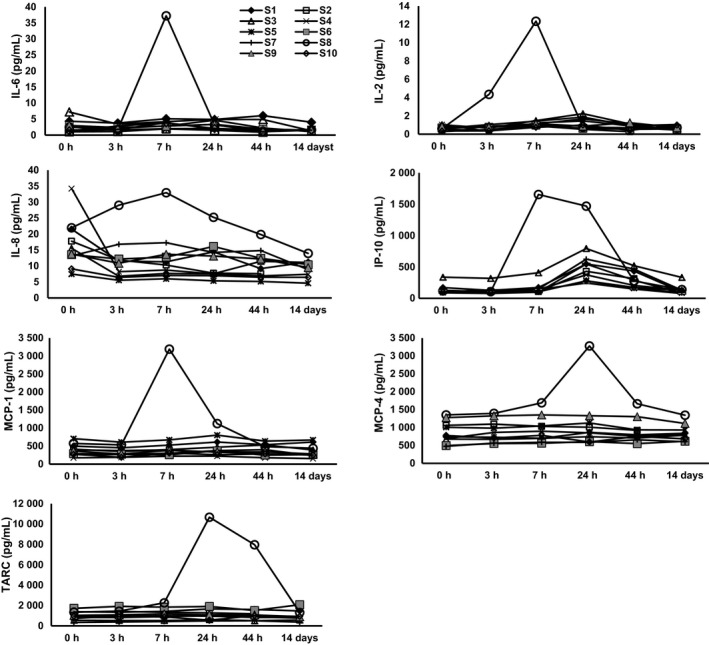
Cytokine profiles in TIV recipients in group 1. Serum cytokine levels in group 1 TIV recipients at 0 h (prior to vaccination), 3 h, 7 h, 24 h, 44 h, and 14 d post‐vaccination. Subject 8 (S8) is shown as open circles. Abbreviations: TIV, trivalent inactivated influenza vaccine; h, hours; d, days.

One subject (subject 8; S8) had a distinctively robust change in IL‐6, IL‐2, IL‐8, IP‐10, and MCP‐1 in response to TIV at 3 and 7 hours and TARC and MCP‐4 at 24 hours (Figure 3).

Although we observed significant changes in CRP, SAA, VCAM‐1, and ICAM‐1 levels within each group, the large amount of variability between the groups precluded the identification of clear patterns of change (data not shown).

### 3.4 Adverse events

Ten subjects reported generalized myalgia after vaccination. Eight were mild in nature; subjects 3 and 8 reported moderate myalgia. The mean time to myalgia onset was 5 hours with a mean duration of 20.7 hours. In subjects who reported myalgia, median serum MCP‐1 levels were greater at baseline and through the first 44 hours post‐vaccination than among those who did not report myalgia (Table 4). Eight subjects reported injection site pain; 7 of them reported mild pain, and subject 8 reported moderate pain. The mean time to pain onset post‐vaccination was 6.25 hours with a mean duration of 21 hours. Pre‐ and post‐vaccination serum IP‐10, IL‐2, IL‐6, IL‐8, and TNF‐α levels tended to be higher, while serum MIP‐1β levels tended to be lower in subjects who reported injection site pain than in those who did not report pain.

**Table 4:** Serum concentration of selected cytokines by time of measurement and by adverse event status

Cytokines that vary based on the presence of myalgia					
	n	Myalgia—present	n	Myalgia—absent	*P*‐value^b^
Hours after vaccination^c^		Median^a^ (IQR)		Median^a^ (IQR)	
MCP‐1					
0 Baseline	10	**404.3 (236.4)**	**10**	**278.4 (145.9)**	**.05**
3	6	413.2 (184.4)	4	2170.4 (48.7)	.06
**7 h post‐vaccination**	**6**	**466.6 (278.0)**	**4**	**280.8 (102.8)**	**.02**
44 h post‐vaccination	10	442.2 (421.3)	10	346.1(103.9)	.08

Cytokines that vary based on the presence of injection site pain					
	n	Injection site pain—present	n	Injection site pain—absent	*P* ‐value
		Median (IQR)		Median (IQR)	
IP‐10					
**0 h post‐vaccination**	**8**	**148.7 (130.6)**	**12**	**108.2 (61.8)**	**.03**
3 h post‐vaccination	4	128.6 (109.0)	6	99.5 (17.1)	.06
**7 h post‐vaccination**	**4**	**289.2 (870.8)**	**6**	**107.3 (36.3)**	**.01**
44 h post‐vaccination	8	584.9 (535.0)	12	313.6 (193)	.24
MIP‐1					
0 h post‐vaccination	8	108.2 (132.2)	12	149.5 (90.5)	.19
**3 h post‐vaccination**	**4**	**111.8 (23.6)**	**6**	**161.9 (102.1)**	**.02**
7 h post‐vaccination	4	123.4 (89.9)	6	164.3 (146.1)	.52
44 h post‐vaccination	8	146.7 (83.8)	12	193.6 (116.2)	.22
IL‐2					
0 h post‐vaccination	8	0.7 (0.3)	12	0.6 (0.6)	.51
**3 h post‐vaccination**	**4**	**1.0 (1.9)**	**6**	**0.5 (0.4)**	**.03**
**7 h post‐vaccination**	**4**	**1.45 (5.6)**	**6**	**1.0 (0.3)**	**.03**
44 h post‐vaccination	8	0.9 (1.1)	12	0.9 (0.6)	.31
IL‐6					
**0 h post‐vaccination**	**8**	**3.0 (3.7)**	**12**	**1.7 (1.6)**	**.04**
3 h post‐vaccination	4	3.1 (1.3)	6	1.9 (0.5)	.09
**7 h post‐vaccination**	**4**	**4.7 (17.1)**	**6**	**2.4 (0.8)**	**.01**
44 h post‐vaccination	12	3.6 (3.1)	8	2.5 (1.0)	.35
IL‐8					
0 h post‐vaccination	8	17.3 (7.3)	12	10.7 (7.9)	.06
3 h post‐vaccination	4	14.2 (13.8)	6	9.6 (5.4)	.20
7 h post‐vaccination	4	14.3 (15.7)	6	9.5 (5.7)	.20
**44 h post‐vaccination**	**8**	**14.3 (5.7)**	**12**	**8.3 (7.6)**	**.05**
TNF**‐**α					
**0 h post‐vaccination**	**8**	**10.1 (6.5)**	**12**	**8.6 (2.0)**	**.05**
3 h post‐vaccination	4	10.2 (5.9)	6	7.5 (2.4)	.06
7 h post‐vaccination	4	12.1 (9.4)	6	7.0 (3.1)	.39
44 h post‐vaccination	8	10.9 (4.0)	12	8.6 (2.0)	.09

a Values in pg/mL.

b *P* ‐value determined using Wilcoxon rank sum test. Bold text for *P*‐values ≤ .05.

c Data at 3 and 7 h are only group 1 (n = 10) and the data at 0 and 44 h are both groups 1 and 2 (n = 20).

In addition, subject 2 (who had reported injection site pain and myalgia) also reported 2 episodes of mild diaphoresis. The first started 14.5 hours after vaccination and lasted 7 hours; the second began 38.5 hours after vaccination and lasted 15 minutes. Subject 3, who also had myalgia, complained of sore throat starting 14.4 hours after receiving the vaccine and lasting for 16 hours. Subject 4 had one episode of vomiting 20 hours after vaccination with no other associated symptoms. Subject 15 had a vasovagal event during the blood draw prior to immunization. Subjects experiencing myalgia had higher levels of IP‐10 and IL‐6 at 7 hours after vaccination (Table 4). Subject 8, as mentioned above, had both moderate injection site pain and moderate myalgia.

## 4 DISCUSSION

Although seasonal influenza vaccines remain the best option to reduce the risk of infection, vaccine efficacy remains controversial, and as of June 2016, the Centers for Disease Control and Prevention (CDC) no longer recommends the use of the live attenuated influenza vaccine (LAIV) more than a decade after its approval in the United States.^23^, ^24^ Ongoing efforts to develop more effective influenza vaccines have had limited success. In addition, the unexpected association of an adjuvanted 2009 H1N1 vaccine in Europe with increased risk of narcolepsy in children^25^, ^26^, ^27^ highlights the need to better understand the biologic mechanisms mediating vaccine responses.

Several recent studies have focused on characterizing the early phase of the immune response in the first days (days 1‐3) after influenza vaccination.^17^, ^18^, ^19^ RNA‐seq profiling identified gene expression patterns predictive of immunogenicity^17^ and adverse events.^18^ Meanwhile, another report identified a correlation between injection site soreness and serum cytokines 1‐2 days after receiving TIV in healthy pregnant and non‐pregnant women.^19^

In our study, we extend our analysis into the first hours following TIV administration and identified temporal patterns of serum cytokine and chemokine changes which occurred as early as 3 hours post‐immunization, generally peaking at approximately 24 hours.

We observed significantly elevated levels of IFN‐γ and IP‐10 beginning at 7 hours and remained elevated at 24 and 44 hours after vaccination, respectively (Figure 2). In addition, we also found that serum IL‐8 levels were reduced after 44 hours and remained so for up to 14 days.

It is important to acknowledge the limitations of this study. The relatively small number of subjects included in this report represents a weakness of our study. Also, we did not perform power calculations to guide sample size estimates. As a result, we cannot be sure whether the study had a sufficient number of subjects to detect the effects of vaccination. In addition, without a sham vaccine and/or no vaccine control group, we cannot effectively control for the effects of injection and/or time on the results. Thus, the results should be interpreted cautiously. Nonetheless, despite the exploratory nature of this study, our findings are largely in agreement with other studies. For example, our observation that serum IFN‐γ and IP‐10 (CXCL10) levels were both elevated 1 day after vaccination was also reported in volunteers given a monovalent 2009 H1N1 vaccine.^18^ In that study, IP‐10 was the only soluble marker associated with adverse events.

Furthermore, the decreased levels of IL‐8 we reported here replicate our previous findings from a larger independent cohort.^22^ Interestingly, Christian et al also found that TIV resulted in decreased serum IL‐8 levels in non‐pregnant women.^28^ As mentioned, the same group followed side effects and serum cytokines daily for 3 days after TIV vaccination in pregnant and non‐pregnant women. They found that at baseline, women who reported more arm soreness had lower IL‐6 and IL‐8 levels and higher IL‐1‐β than those that did not, and those women also had higher TNF‐α and macrophage migration inhibitory factor (MIF) levels in the days following vaccination.^19^ Although we did not measure MIF, the other group did not assess IP‐10, and our cohorts are rather different, we did find that volunteers with injection site pain had higher IP‐10 and IL‐6 levels at baseline and after vaccination, as well. In addition, we noted that volunteers who experienced myalgia had elevated MCP‐1 levels at baseline as well as 3 hours and 7 hours after vaccination compared to those who did not report myalgia (Table 4). We did not however see a statistically significant change in IL‐8 between those with symptoms and those without, although that may be due to our small sample size.

Like others, we observed considerable interindividual variation in the levels of these markers of inflammation which could explain in part the variability in reported symptoms following influenza vaccination.^29^ Although our power to detect effects was somewhat restricted by the sample size, we noted far less intra‐individual variability in that subjects with higher median levels tended to remain higher while subjects with lower median levels tended to remain lower.

Taken together, our data support a growing body of literature indicating that soluble markers of inflammation may serve as a much‐needed early indicator of vaccine reactogenicity and risk for adverse events.^18^ Importantly, our findings indicate that peripheral cytokines begin to change in the hours immediately following vaccination and warrant further exploration in larger studies to determine the biologic basis of clinical symptoms associated with vaccination. Improving our basic understanding of the immune response(s) to vaccination may enhance influenza vaccine development efforts and public health safety.

## CONFLICT OF INTEREST

Neal Halsey has received an honorarium from Pfizer for a one‐day meeting and he serves of safety monitoring board for Takeda for an unrelated experimental vaccine. The other authors report no conflict of interests.
